# A standardized imaging and analysis workflow for quantitative evaluation of cutaneous neurofibromas in *Nf1*-KO mice

**DOI:** 10.1371/journal.pone.0354818

**Published:** 2026-07-27

**Authors:** Laura Fertitta, Fanny Coulpier, Layna Oubrou, Xavier Decrouy, Etienne Audureau, Nicolas Ortonne, Pierre Wolkenstein, Piotr Topilko

**Affiliations:** 1 Department of Dermatology, Henri Mondor University Hospital, Assistance Publique – Hôpitaux de Paris (AP-HP), Referral center for Neurofibromatosis (CERENEF), Creteil, France; 2 Inserm, Clinical Investigation Center 1430, Henri Mondor University Hospital, AP-HP, Creteil, France; 3 STEM REPAIR NF Team, Inserm U955, Mondor Institute for Biomedical Research (IMRB), Creteil, France; 4 Imaging Plateform, Mondor Institute for Biomedical Research (IMRB), Creteil, France; 5 Public Health Department, Henri Mondor University Hospital, AP-HP, Creteil, France; 6 Université Paris Est Créteil (UPEC), Créteil, France; 7 Department of Pathology, Henri Mondor University Hospital, AP-HP, Creteil, France; University of Campania Luigi Vanvitelli: Universita degli Studi della Campania Luigi Vanvitelli, ITALY

## Abstract

Neurofibromatosis type 1 (NF1) is an autosomal dominant disorder in which cutaneous neurofibromas (cNFs) represent one of the most common and burdensome manifestations. No approved pharmacological treatment exists. Preclinical studies are essential to evaluate candidate therapies, but reliable outcome and endpoint measures for cNFs in animal models remain limited. We developed and validated a standardized methodology to assess drug efficacy in the *Prss56Cre Nf1-KO* mouse model which recapitulates key features of cNFs. In this model, *Nf1* inactivation and tdTomato (Tom) reporter expression were specifically targeted to Schwann cells (SCs) responsible for cNF development. This approach enables real-time monitoring, isolation, and manipulation of tumor SCs at any time. We defined macroscopic (tumor count, total Tom^+^ fluorescent surface area, fluorescence intensity) and microscopic (cell-type composition defined by immunolabeling with a panel of specific markers, area quantification) endpoints, developed dedicated ImageJ scripts for automated image analysis, and compared the results with those obtained using the conventional manual method. Both automated measurements showed excellent reproducibility (ICC = 1) and strong correlation with manual analysis (Spearman’s coefficient > 0.90), while significantly reducing analysis time (up to 100-fold faster). Bland–Altman analyses confirmed the absence of systematic bias compared with manual scoring. The standardized image naming and metadata integration further facilitated data consolidation and statistical analysis. This validated approach provides a reliable, reproducible, and time-efficient framework for evaluating drug effects on cNFs in preclinical studies. It establishes a foundation for robust efficacy testing of candidate therapies, facilitates cross-study comparability, and accelerates therapeutic development and clinical translation.

## Introduction

Neurofibromatosis type 1 (NF1) is an autosomal dominant disorder with an incidence of approximately 1 in 2500 individuals [1–4]. It arises from a germline heterozygous loss-of-function mutation in the tumor suppressor *NF1* gene, which encodes neurofibromin, a negative regulator of the RAS signaling pathway [5]. Somatic inactivation of the second allele causes a total loss of neurofibromin function, which in turn leads to RAS pathway hyperactivation and the emergence of the disorder’s wide range of clinical manifestations [6]. Although NF1 can affect nearly every organ, cutaneous manifestations are the most common and often serve as key diagnostic criteria [7]. Among these, benign peripheral nerve sheath tumors known as cutaneous neurofibromas (cNFs) occur in the vast majority of individuals with NF1 [8]. These tumors can profoundly impact quality of life (QoL) [9,10], primarily due to physical disfigurement [11]. They may arise anywhere on the skin and can number in the thousands throughout a patient’s lifetime. Currently, treatment options are limited to destructive or ablative procedures.

Clinically, cNFs exhibit a broad spectrum of appearances, ranging from barely visible violaceous macules (nascent or latent cNFs) to raised lesions, which may present as flat, sessile, globular or pedunculated soft masses [12,13]. Histologically, these tumors are non-encapsulated and consist of both a cellular component and a rich extracellular matrix (ECM). The cellular population includes NF1^-/-^ tumor Schwann cells (SCs), as well as elements of the tumor microenvironment such as dermal and nerve sheath fibroblasts (FBs), immune cells, blood vessels and axons [13–15]. SCs with biallelic *NF1* loss are considered as population of origin, responsible for cNFs development [16–18], although the exact mechanism of tumorigenesis remains unclear. The ECM contains variable amounts of different collagen types [19,20].

While several *in vitro* and animal models of cNFs have been developed and characterized [21], few accurately recapitulate the full complexity of these tumors. Our laboratory has generated and characterized a *Prss56Cre Nf1-*KO mouse model that faithfully reproduces several NF1 manifestations, including cNFs [22]. In this model, biallelic *Nf1* loss and expression of the fluorescent reporter tdTomato (Tom) are simultaneously induced in *Prss56*-expressing cells, such as boundary cap cells, at the origin of SCs populating nerve roots and nerve endings of skin, two locations where NFs often develop in patients. Although the histological composition of murine cNFs closely resembles that observed in patients, their macroscopic appearance differs. Mouse cNFs are flat and punctate, making them difficult to see with the naked eye. However, their visualization in live animals is possible using epifluorescence, thanks to the Tom reporter. Of note, skin trauma accelerates cNF development in this model, enabling the generation of calibrated cohorts with numerous tumors necessary for preclinical drug testing [23].

Even though *in vitro* models, including organoids, are increasingly being developed, the use of dedicated animal models remains an essential component for basic and translational research: clinical trials must always be preceded by comprehensive pharmacological and toxicological testing in animals [24,25]. Nevertheless, scientists are increasingly aware of their limitations, and their predictive power should be interpreted with caution [26–28]. Moreover, unlike safety assessments, efficacy evaluation lacks formalized guidance, as each new drug requires a customized approach based on its mechanism of action and therapeutic indication [29,30].

Because each preclinical model should correspond to a defined therapeutic indication and be evaluated using rigorous methodology, we sought to design and validate a standardized preclinical workflow, together with robust efficacy endpoints, for drugs intended to prevent or reduce cNFs in our mouse model. Ultimately, this work aims to provide the scientific community with the necessary tools to enable reliable use of this model for cNF therapeutic evaluation.

## Materials and methods

The study was structured into three consecutive phases.

### 1. Experimental design definition

Study design, endpoints, corresponding outcomes, and measurement methods were established. Macroscopic and pathological endpoints of cNFs in the *Prss56Cre Nf1-*KO mouse model were defined based on model evaluation modalities: (i) 2D photographs (.tif format) acquired with LasX® software using a fluorescent magnifying loupe (Leica®) enabling visualization of Tom expression, and (ii) 2D photographs of immunofluorescence (IF) 14 *μm* slides (.czi format) acquired with a confocal microscope (Zeiss® LSM 900). Two experimental designs were defined, each incorporating specific exposure times for macroscopic image acquisition: a preventive protocol (assessing a drug’s ability to prevent/delay cNF development) and a curative protocol (assessing a drug’s ability to shrink existing cNFs).

In this study, no animal experiments were performed. Only macroscopic and IF images from previous studies [23] approved by the Institutional Animal Care and Use Committee (reference number: APAFIS#25551-2020031115016451 v6) were used. In these previous studies, animals were housed in groups (3-5 siblings/cage) to generate cNF as previously described [23,31]. Of note, skin microtraumas were not induced manually but resulted from the innately aggressive behavior of group-housed male mice; these lesions were barely visible to the naked eye. Mice were daily monitored from birth, and sacrificed upon reaching the endpoint criteria of the corresponding study or if critical animal welfare endpoints were identified. Following sacrifice, back skin was dissected, cNFs were collected and processed for further analyses [23].

### 2. Development of an automated outcome measurement method

To ensure reproducibility of outcome measures, dedicated ImageJ (https://imagej.net/software/fiji/) scripts were developed for the automated analysis using 10 macroscopic and 10 IF 2D photographs from previous studies, with the guidance of an image analysis expert (XD).

These scripts were designed to quantify Tom fluorescence on the dorsal skin of the animal (macroscopic outcomes) as well as histological composition of tumors (IF outcomes), including SCs, FBs, immune cells, and ECM. IF was performed as previously described [23] using an antibody panel validated in this model (S1 Table).

### 3. Validation of the outcome measurement tools

The automated scripts were validated following methodologies usually applied to clinician-reported outcome measures in humans. This phase focused on Tom-related outcomes (macroscopic and IF). Images from previous studies (different from phase 2) were analyzed both automatically using the dedicated scripts and manually by two blinded investigators using a Wacom® Cintiq tablet. Reproducibility of both automated and manual methods was evaluated using the intraclass correlation coefficient (ICC): (i) analysis of the same images using the scripts on two different computers, and (ii) analysis of the same images by two blinded investigators. A total of 50 macroscopic (25 from systemic treatment studies and 25 from topical) and 25 IF images were included to ensure sufficient statistical power. An ICC > 0.8 was considered acceptable [32]. Automatically generated outcomes were compared with manual measurements (average values from two blinded investigators) using Spearman’s rank correlation coefficients. Agreement between automated and manual methods was assessed with Bland–Altman plots [33].

The mean time required to analyze an image manually (*μ*m) or automatically (*μ*a) was measured and compared using either Student’s t-test or the Wilcoxon test, depending on the normality of the distribution. Statistical analyses were performed with RStudio 1.4.1717 (R Software Inc., Delaware, USA). All data supporting the conclusions of this study are publicly available through the Synapse repository and can be accessed at https://doi.org/10.7303/syn51667227 (accession syn51471542).

## Results

### 1. Experimental design definition

Two protocols for preclinical studies, in compliance with current regulations [34], were established. In the curative protocol, the candidate compound (administered either systemically or topically) was introduced in mice already presenting numerous mature cNFs. Their development was triggered by housing several males together for one to two months, followed by a one-month separation to allow skin inflammation to subside and wounds to heal – given their naturally aggressive behavior – before starting treatment. In the preventive protocol, the molecule (systemic or topical) was delivered to younger mutant males (average age 2–4 months), prior to the onset of tumors. Mice were housed together during the treatment phase.

For both protocols, the same macroscopic outcomes were evaluated at baseline and at the end of treatment, comparing (i) the treated versus placebo groups (inter-group comparison) and (ii) changes within each group from baseline to endpoint (intra-group comparison). For topical treatments, one skin area was defined for drug application and another for placebo/vehicle. Macroscopic image analysis was then performed following the selection (S1 Fig) of these regions of interest. Post-mortem IF was used to compare cNF skin samples from treated and placebo groups. Three macroscopic endpoints of cNFs were defined based on Tom fluorescence visualized with the fluorescent magnifying macro zoom: (i) the number of cNFs, (ii) the total fluorescent surface area (TFSA), and (iii) the fluorescence intensity. Two IF endpoints were defined according to the antibody panel used: (i) the estimated proportion of specific cell types and (ii) the estimated proportion of the area of interest. Each generated image was required to follow a standardized naming convention, with identifiers adapted accordingly: for macroscopic images, “YYYY MM DD_drugname_beforeorafter_mousenumber”, and for IF images, “YYYY MM DD_drug_mousenumber_cNFnumber_PANEL-DAPI-TOM”. This allowed for the standardization of terminology in the CSV tables generated by automated analysis in ImageJ and facilitated the consolidation of all data into a single metadata table (using RStudio).

### 2. Development of an automated outcome measurement method

The macroscopic and IF endpoints were translated into their corresponding outcomes on ImageJ, enabling the generation of two dedicated scripts: one for macroscopic analysis (full ImageJ script provided in S2 Fig) and one for IF analysis (full ImageJ script provided in S3 Fig). For each IF image series, prior to initiating automated analysis, the user only needed to adapt the script by: i) adjusting the contrast threshold of the images, since this parameter varies with the acquisition modalities and instruments and cannot be perfectly standardized across studies (line 80 for DAPI and line 101 for Tom, S3 Fig), and ii) specifying the number and order of channels (e.g., C1, C2, C3) according to the sequence of image acquisition. The script for IF image analysis was adapted for all antibodies described in S1 Table.

### 3. Validation of the outcome measurement tools

The reproducibility of the automated method was perfect (ICC = 1) for each type of pictures. The inter-rater reproducibility was considered as good for macroscopic outcomes and moderate for the IF outcomes (Table 1).

**Table 1:** Inter-rater reliability of manual macroscopic and immunofluorescence outcomes using the Intraclass Correlation Coefficient (ICC), and their 95% confidence interval (95%CI).

Outcomes	ICC [95%CI]
Macroscopic fluorescence intensity (mean)	1
Total fluorescent surface area	0.862 [0.769; 0.919]
Number of cutaneous neurofibromas	0.926 [0.874; 0.957]
IHC fluorescence intensity (mean)	1
Tomato+ area	0.631 [0.322; 0.819]
Number of Tomato+ Schwann cells	0.727 [0.473; 0.870]

The Spearman coefficients were of 0.92 for number of cNF, 0.93 for TFSA and 1 for fluorescent intensity. They were of 0.92 for the number of Tom^+^ SCs and of 0.93 for the Tom^+^ area.

**Fig 1:**
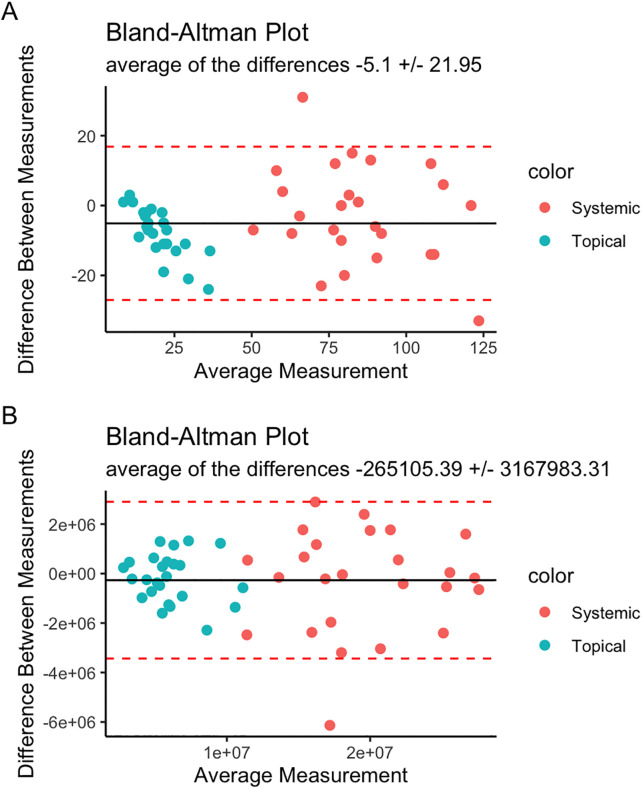
Bland-Altman plots of macroscopic image analysis results. (A) Number of cutaneous neurofibromas; (B) Total fluorescent surface area.

**Fig 2:**
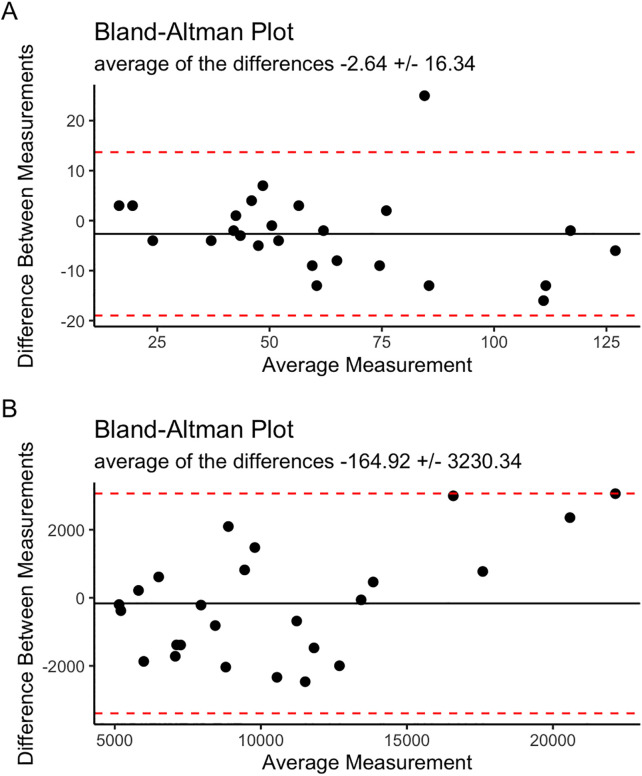
Bland-Altman plots of macroscopic image analysis results. (A) Number of tdTomato^+^ Schwann cells; (B) TdTomato^+^ area.

The Bland-Altman plots showed no systematic biased of measurement of macroscopic (Fig 1) and IF (Fig 2) outcomes and a maximum of average of differences between the measurements generated through the manual and the automated methods of 9% for the number of cNF with an under-estimation of the manual counting. The average of differences represented 1% of the TFSA, 0.5% of the Tom+ area and 4% of the number of Tom^+^ SC.

The mean analysis time per image was significantly reduced with the automated (*μ*a) method compared to manual (*μ*m): for macroscopic images from systemically treated mice, *μ*m = 3.22 ± 0.49 min vs. *μ*a = 0.06 ± 0 min (p < 0.001, IC95%[2.56;3.18]]); for topically treated mice, *μ*m = 1.85 ± 0.23 min vs. *μ*a = 0.03 ± 0 min (p < 0.001, IC95%[1.52;1.80]); and for IF images, *μ*m = 9.24 ± 1.35 min vs. *μ*a = 0.03 ± 0 min (p < 0.001, IC95%[8.96;10.24]).

## Discussion

In this study, we designed and validated a robust method for evaluating the efficacy of drugs on cNFs in the *Prss56Cre Nf1-KO* mouse model, both macroscopically and by IF. This approach can be applied to preclinical studies targeting cNFs in this model, thereby improving reproducibility, enabling cross-study comparability, and accelerating therapeutic development and clinical translation.

Our method, based on 2D macroscopic imaging and IF analysis, is reliable (Spearman coefficient >0.90), reproducible (ICC = 1), and time-efficient for researchers using this model. Although the *Prss56Cre Nf1-KO* mouse model has already been successfully used in preclinical trials over the past few years [23,35], future studies on cNFs can now benefit from this standardized evaluation method. Moreover, the use of standardized terminology also facilitates the creation of metadata tables, enabling subsequent statistical analyses. Finally, aligning outcomes and evaluation tools, such as the proposed method, for studying the mouse model will enable comparisons between studies and thereby accelerate the identification of compounds of interest. To further enhance cNF analyses in this model, we are evaluating a highly sensitive, benchtop *in vivo* optical imaging system (φ-eye™, Bioemtech®) capable of fluorescence and bioluminescence detection and simultaneous imaging of three live mice.

Several NF1 animal models, including mice and minipigs [36,37], have been developed to investigate NF1-related cNFs [21]. Two additional mouse models – *HoxB7Cre Nf1-KO* [38] and *Sox10Cre Nf1-KO* [39] – have been of interest for studying cNFs. While each model has its limitations, the *Prss56Cre Nf1-*KO mouse model presents the specific clinical feature that cNFs are flat and therefore not measurable with conventional tools such as rulers or calipers. Although we initially considered developing and validating a subjective scoring system (e.g., continuous or Likert-type scales), we determined that an objective method with minimal bias was preferable.

Regarding IF image analysis, we consider it indispensable for interpreting tumor responses to treatment, particularly since tumor SCs in our model express the fluorescent Tom reporter. While complementary validation methods (e.g., FACS, Western blotting, transcriptomic analysis) may be applied, the ability to semi-quantify IF images provides an additional tool for assessing the effect of candidate compounds.

Over the past decade, several initiatives have been launched to improve the design and reporting standards of preclinical studies [40–42], including the more recent European Quality in Preclinical Research (EQIPD) initiative, supported by the European Union’s Innovative Medicines Initiative. EQIPD aims to foster innovation by ensuring the generation of robust and reliable preclinical data [43]. Ferreira *et al.* also developed a tool to assess, validate, and compare the clinical translatability of animal models used for preliminary evaluation of drug efficacy. The Framework to Identify Models of Disease (FIMD) comprises a questionnaire spanning eight domains and generates a score out of 100. It aids drug development by guiding the selection of the most relevant disease model, thereby increasing the likelihood of producing meaningful and translatable results to advance drug candidates into clinical testing [44]. Finally, proper assessment of the external validity of efficacy models – that is, the extent to which results from nonhuman animals can be generalized to humans – is now widely recognized as a critical factor for successful translation [27]. Together, these initiatives aim to minimize the biases inherent to animal experimentation, ensuring that the results of preclinical studies are reliable, reproducible, and translatable to humans.

Alongside the identification of therapeutic targets and preclinical studies in animal models, clinical trials targeting cNFs are being prepared. Indeed, the penetrance of NF1 reaches 97% by 8 years of age and 100% after 20 years [45], with cNFs affecting nearly all individuals [8]. Although these tumors carry no risk of malignant transformation, they can substantially impair quality of life [9,10], particularly due to physical disfigurement [11]. Recently, the international Response Evaluation in Neurofibromatosis and Schwannomatosis (REiNS) consortium defined a core outcome domain set suitable for clinical trials targeting NF1-related cNFs [46] and developed dedicated measurement tools. In addition, within the European Patient-Centric Clinical Trial Platforms (EU-PEARL), innovative platform-basket trials have been designed for NF1, increasing the likelihood of rapidly identifying effective treatments at lower cost compared with traditional single-agent clinical trials [47]. Together, these essential efforts to standardize each step of drug development, from preclinical studies to human trials, converge toward the most rapid and reliable identification of future therapies.

Our approach presents two major limitations. First, our method has not yet been evaluated for sensitivity to change; its responsiveness can only be determined in future preclinical trials using this workflow. Second, the macroscopic outcome “number of cNFs” should be interpreted with caution. While this parameter is expected to decrease or stabilize with effective therapy – or increase if ineffective – cNFs in our model can merge in cases of therapeutic inefficacy, potentially resulting in a falsely reduced count. For macroscopic analyses, TFSA and fluorescence intensity are therefore preferred measures.

In conclusion, we have developed and validated a workflow that integrates macroscopic and immunofluorescence image analysis and quantification, providing a reliable, reproducible, and time-efficient framework for evaluating drug effects on cNFs in preclinical studies. This approach lays the foundation for robust efficacy testing, cross-study comparability, and the development and clinical translation of candidate therapies.

## Supporting information

S1 Table List of antibodies for assessing cutaneous neurofibromas in the *Prss56*^Cre^ *Nf1-*KO mouse model. (DOCX)

S1 Fig Automated region-of-interest analysis of Tomato fluorescence in mouse dorsal skin after topical treatment. Representative 2D photograph of mouse back skin following topical treatment. Images were acquired using a fluorescent magnifying loupe (Leica Microsystems) enabling visualization of tdTomato reporter expression and analyzed with LAS X software. The region of interest used for automated analysis is highlighted in red.
(TIFF)

S2 Fig ImageJ script for automated analysis of macroscopic images. (TIFF)

S3 Fig ImageJ script for automated analysis of immunofluorescence images. (TIFF)
