# Supplementary material for: A standardized imaging and analysis workflow for quantitative evaluation of cutaneous neurofibromas in Nf1-KO mice
Source: PLoS One. 2026 Jul 27;21(7):e0354818. doi: 10.1371/journal.pone.0354818 (PMC13405064; doi:10.1371/journal.pone.0354818)
Supplement: S1 Table — (DOCX) [file pone.0354818.s001.docx]

**Supplementary table 1: List of antibodies for assessing cutaneous neurofibromas in the *Prss56*^Cre^ *Nf1-*KO mouse model.** The samples were sectioned to 14μm thickness on Super Frost Plus slides. Immunofluorescence was performed on mouse frozen sections using the following primary antibodies.

| Component | Target | Species | Dilution | Reference |
| --- | --- | --- | --- | --- |
| Tumor Schwann cells | Tomato | Rat | 1/1000 | Est203 |
|  |  | Rabbit | 1/1000 | 600-401-379 |
| Fibroblasts | PDGFR$\alpha$ | Goat | 1/100 | AF1062 |
| MAPK pathway activity | Phospho-ERK | Rabbit | 1/250 | 71-7800 |
| Immune cells | CD45 | Goat | 1/100 | AF114 |
| Macrophages | F4/80 | Rat | 1/400 | Ab6640 |
| Fibrosis | Periostin | Goat | 1/400 | AF1062 |
|  | Collagen 1A | Rabbit | 1/200 | CL5015AP-1 |
| Proliferation | Ki67 | Rabbit | 1/200 | Ab15580 |
| Vascularization | PECAM (CD31) | Goat | 1/500 | AF3628 |
| Innervation | PGP9.5 | Rabbit | 1/100 | Ab108986 |
