# Supplementary figures and images for: A standardized imaging and analysis workflow for quantitative evaluation of cutaneous neurofibromas in Nf1-KO mice
Source: PLoS One. 2026 Jul 27;21(7):e0354818. doi: 10.1371/journal.pone.0354818 (PMC13405064; doi:10.1371/journal.pone.0354818)

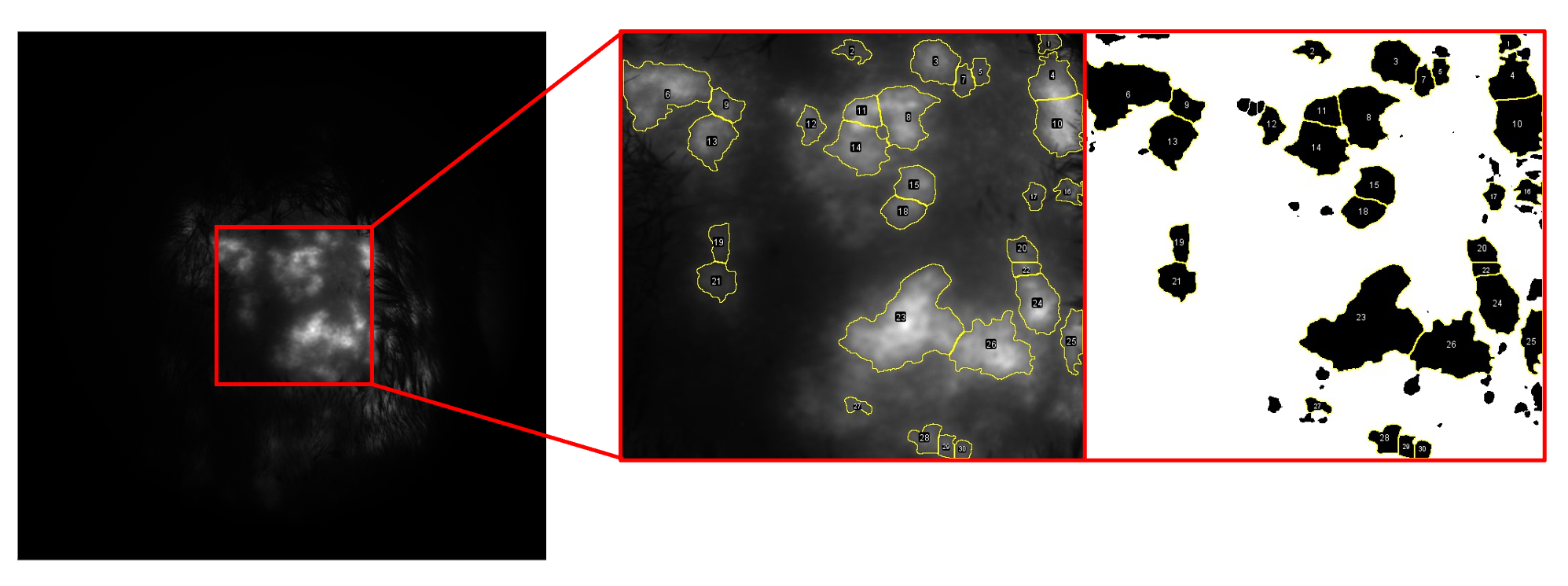

Supplement: S1 Fig — Representative 2D photograph of mouse back skin following topical treatment. Images were acquired using a fluorescent magnifying loupe (Leica Microsystems) enabling visualization of tdTomato reporter expression and analyzed with LAS X software. The region of interest used for automated analysis is highlighted in red. (TIFF) [file pone.0354818.s002.tiff]
